# The Dental Review

**Published:** 1887-02

**Authors:** 


The Dental Review, of Chicago, is a new aspirant for professional favor, the first number having appeared in November, 1886. Its editors are Drs. Harlan, Wassel, Reid, Ottofy, and Davis, and it bids fair to be a valuable contribution to dental periodical literature.

